# Insulin-like Growth Factor Signaling Promotes Corneal Myofibroblast Survival and Sustains Corneal Fibrosis

**DOI:** 10.3390/ijms27167320

**Published:** 2026-08-16

**Authors:** Yunjeong Hwang, Kyung-No Son, Manoj Chaudhary, Eunbee Lee, Minhyung Kim, Sungyong You, Terry J. Smith, Vinay Kumar Aakalu, Kyu-yeon Han

**Affiliations:** 1 Department of Ophthalmology and Visual Sciences, Illinois Eye and Ear Infirmary, College of Medicine, University of Illinois Chicago, Chicago, IL 60612, USA; yjhwang1@uic.edu (Y.H.); elee237@uic.edu (E.L.); 2 Richard and Loan Hill Department of Biomedical Engineering, University of Illinois Chicago, Chicago, IL 60612, USA; 3 Department of Ophthalmology and Visual Sciences, Eye Institute, Medical College of Wisconsin, Milwaukee, WI 53226, USA; kson@mcw.edu (K.-N.S.); mchaudhary@mcw.edu (M.C.); 4 Departments of Urology and Computational Biomedicine, Cedars-Sinai Medical Center, Los Angeles, CA 90048, USA; minhyung.kim@cshs.org (M.K.); sungyong.you@csmc.edu (S.Y.); 5 Department of Internal Medicine, University of Michigan Medical School, Ann Arbor, MI 48105, USA; terrysmi@med.umich.edu; 6 Department of Ophthalmology and Visual Sciences, Kellogg Eye Center, University of Michigan, Ann Arbor, MI 48105, USA

**Keywords:** corneal fibrosis, Insulin-like growth factor, linsitinib

## Abstract

Corneal fibrosis is a major cause of visual impairment worldwide and is characterized by persistent stromal scarring that disrupts corneal transparency. Although transforming growth factor-β (TGF-β)-mediated myofibroblast differentiation is well established, the mechanisms that sustain myofibroblast survival and persistence remain poorly understood. In this study, we investigated the role of the insulin-like growth factor (IGF) signaling axis in corneal myofibroblast survival and evaluated the therapeutic potential of linsitinib, a dual IGF-1 receptor (IGF-1R)/insulin receptor (INSR) inhibitor. Primary corneal myofibroblasts were exposed to pro-apoptotic conditions and treated with IGF ligands in the presence or absence of linsitinib. The effects of IGF pathway inhibition were further examined in a mouse model of corneal fibrosis. IGF-1 and IGF-2 promoted myofibroblast survival under pro-apoptotic conditions, whereas pharmacologic inhibition of IGF-1R/INSR signaling with linsitinib blocked these pro-survival effects. In vivo, linsitinib treatment reduced myofibroblast persistence and attenuated corneal fibrosis. These findings identify the IGF axis as a critical regulator of corneal myofibroblast survival and suggest that persistent fibrosis is maintained, in part, by IGF-dependent resistance to apoptosis. Targeting survival pathways rather than myofibroblast differentiation may represent a novel therapeutic strategy for the treatment of corneal fibrosis.

## 1. Introduction

Corneal fibrosis is a leading cause of visual impairment worldwide and remains a major clinical challenge. Loss of corneal transparency due to stromal scarring disrupts light transmission and degrades visual acuity, frequently resulting from chemical injury, infection, surgery, or trauma [1,2,3]. Current therapies primarily target acute inflammation or limit early injury progression but are largely ineffective once myofibroblasts become established and stromal scarring develops [4,5,6]. A better understanding of these mechanisms is needed to identify new therapeutic approaches for fibrotic corneal disease.

Fibrosis in the cornea, as in other organs, is driven by the emergence and persistence of myofibroblasts, specialized contractile cells that deposit extracellular matrix (ECM) and remodel tissue architecture [7,8]. During normal wound healing, myofibroblasts are transient and are eliminated through apoptosis once tissue repair is complete [9,10,11,12]. In pathological conditions, myofibroblasts evade apoptosis and persist within the tissue, resulting in excessive ECM deposition, stromal disorganization, and permanent scarring [13,14,15]. Although the differentiation of fibroblasts into myofibroblasts, largely mediated by transforming growth factor-β (TGF-β), has been extensively investigated [16,17], considerably less is known about mechanisms maintaining myofibroblast survival after differentiation and that allow these cells to persist in fibrotic corneas.

During normal wound healing, physiological withdrawal of profibrotic signals, exposure to pro-apoptotic mediators such as interleukin-1 (IL-1), and restoration of epithelial–stromal homeostasis promote myofibroblast apoptosis [18,19,20,21]. Persistence of myofibroblasts in fibrotic tissue therefore implies activation of survival pathways that counteract apoptotic signaling [22]. Thus, pathways regulating cell survival may play important roles in determining the magnitude and duration of fibrosis. However, molecular mechanisms responsible for maintaining corneal myofibroblast survival remain poorly defined.

Among the signaling pathways implicated in cell survival, the insulin-like growth factor (IGF) axis is particularly notable because of its potent anti-apoptotic activity and its established role in tissue repair and wound healing [23,24,25]. Binding of IGF-1 or IGF-2 to IGF receptor (IGF-1R), insulin receptor (INSR), or hybrid (heterodimeric) IGF-1R/INSR activates downstream signaling pathways including PI3K–AKT and MAPK–ERK, which promote cell survival and suppress apoptosis [26,27]. Consistent with these functions, IGF signaling regulates fibroblast behavior and fibrotic remodeling in multiple organ systems, including the lung, liver, kidney, and orbit [28,29,30,31,32,33,34]. Components of the IGF axis are expressed in the cornea, where they contribute to epithelial homeostasis, wound healing, and stromal cell responses following injury [35,36,37]. Nevertheless, whether IGF signaling directly regulates the survival and persistence of differentiated corneal myofibroblasts remains unknown.

Linsitinib (OSI-906) is a selective tyrosine kinase inhibitor of IGF-1R and INSR that was originally developed for the treatment of IGF-driven malignancies [38]. In the present study, linsitinib was used as a pharmacological tool to determine whether IGF-dependent survival pathways contribute to corneal myofibroblast persistence during fibrosis.

Here, we investigated whether the IGF signaling axis functions as a survival pathway for corneal myofibroblasts. Using primary corneal myofibroblasts and a murine model of corneal fibrosis, we investigated whether IGF signaling functions as a survival pathway that promotes myofibroblast persistence. Our findings demonstrate that IGF-1 and IGF-2 enhance myofibroblast survival, whereas pharmacological inhibition of IGF-1R/INSR signaling suppresses survival, thereby attenuating corneal fibrosis in vivo.

## 2. Results

### 2.1. IGF Ligands Modestly Enhance Corneal Fibroblast Proliferation but Do Not Drive Differentiation

**Figure 1:**
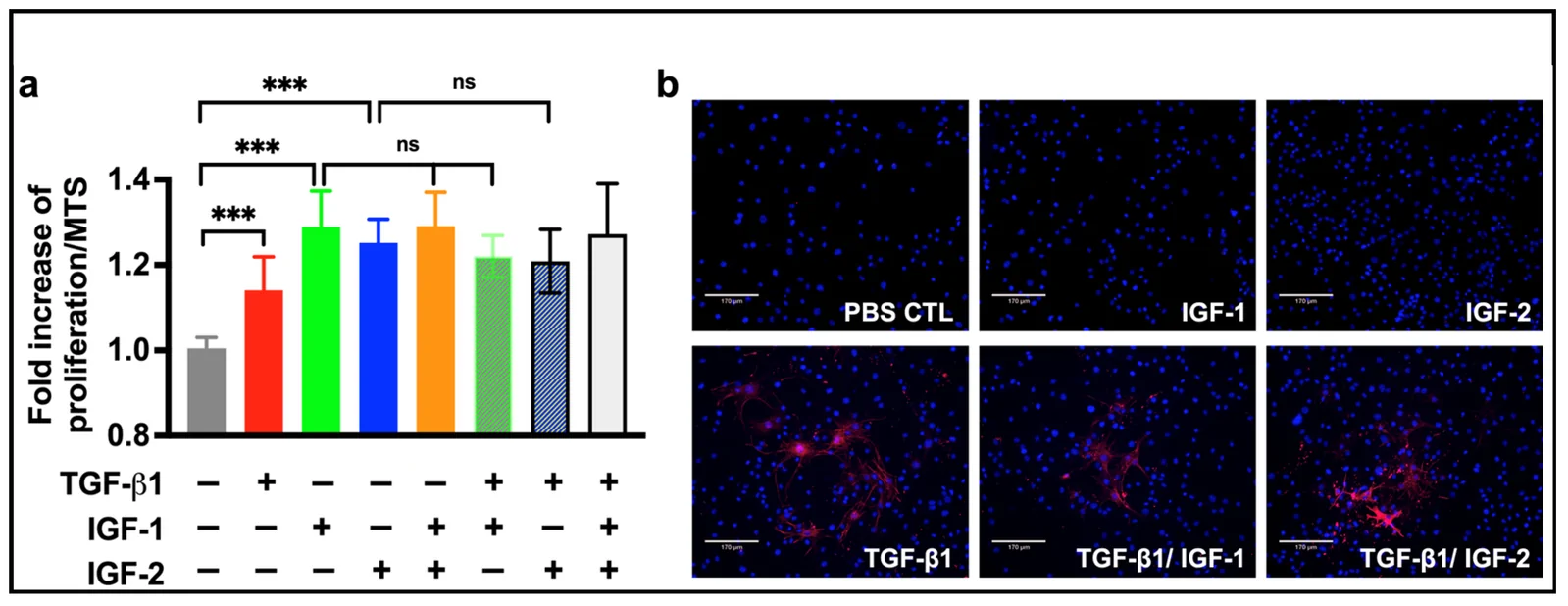
**IGF signaling modestly increases corneal fibroblast proliferative activity without inducing myofibroblast differentiation.** (**a**) Treatment with IGF-1 or IGF-2 (100 ng/mL) significantly increased MTS activity in primary mouse corneal fibroblasts compared with untreated controls. Combined treatment with both ligands failed to further enhance proliferation, and co-treatment with TGF-β1 did not significantly alter TGF-β1-induced proliferative activity. (**b**) Immunofluorescence staining for α-smooth muscle actin (α-SMA; red) demonstrated robust myofibroblast differentiation following TGF-β1 treatment, whereas IGF-1 or IGF-2 alone failed to induce detectable α-SMA expression. Co-treatment with IGF-1 or IGF-2 did not alter TGF-β1-induced α-SMA expression. Nuclei were counterstained with DAPI (blue). Data are presented as mean ± SEM (*n* = 6). Scale bar = 170 μm. *** *p* < 0.001; ns, not significant. Bar colors are used solely to distinguish the treatment groups.

To determine whether IGF signaling influences corneal fibroblast proliferative activity and differentiation, primary mouse corneal fibroblasts were treated with IGF-1 and/or IGF-2 (100 ng/mL) in the presence or absence of TGF-β1 (10 ng/mL). Both IGF-1 and IGF-2 significantly increased proliferation as assessed using an MTS assay compared with untreated controls (Figure 1a). Combined treatment with IGF-1 and IGF-2 did not further increase proliferation. TGF-β1 also increased proliferation, and co-treatment with IGF-1 and/or IGF-2 did not significantly augment the response to TGF-β1 (*n* = 6).

To evaluate whether IGF signaling promotes myofibroblast differentiation, α-smooth muscle actin (α-SMA) expression was assessed by immunofluorescence. As expected, TGF-β1 induced robust α-SMA expression and stress fiber formation, consistent with myofibroblast differentiation (*n* = 3) (Figure 1b). In contrast, neither IGF-1 nor IGF-2 induced detectable α-SMA expression above control levels. Furthermore, co-treatment with IGF-1 or IGF-2 failed to detectably affect TGF-β1-induced α-SMA expression. Similar results were observed following combined treatment with IGF-1, IGF-2, and TGF-β1 (Supplementary Figure S1). These findings indicate that although IGF ligands modestly enhance corneal fibroblast proliferation but fail to induce myofibroblast differentiation or modify TGF-β1-driven differentiation.

### 2.2. Transcriptomic Analyses Identify Activation of the IGF Signaling Pathway During Corneal Myofibroblast Differentiation

To identify molecular pathways associated with corneal myofibroblast differentiation, bulk RNA sequencing was performed on primary mouse corneal fibroblasts treated with TGF-β1 (10 ng/mL) for 3 days and compared with untreated fibroblasts using three independent biological replicates per group (*n* = 3). Multidimensional scaling (MDS) analysis demonstrated clear separation between untreated and TGF-β1-treated samples, with the first dimension accounting for 94% of the total variance, indicating excellent reproducibility and minimal variability among biological replicates (Supplementary Figure S2).

**Figure 2:**
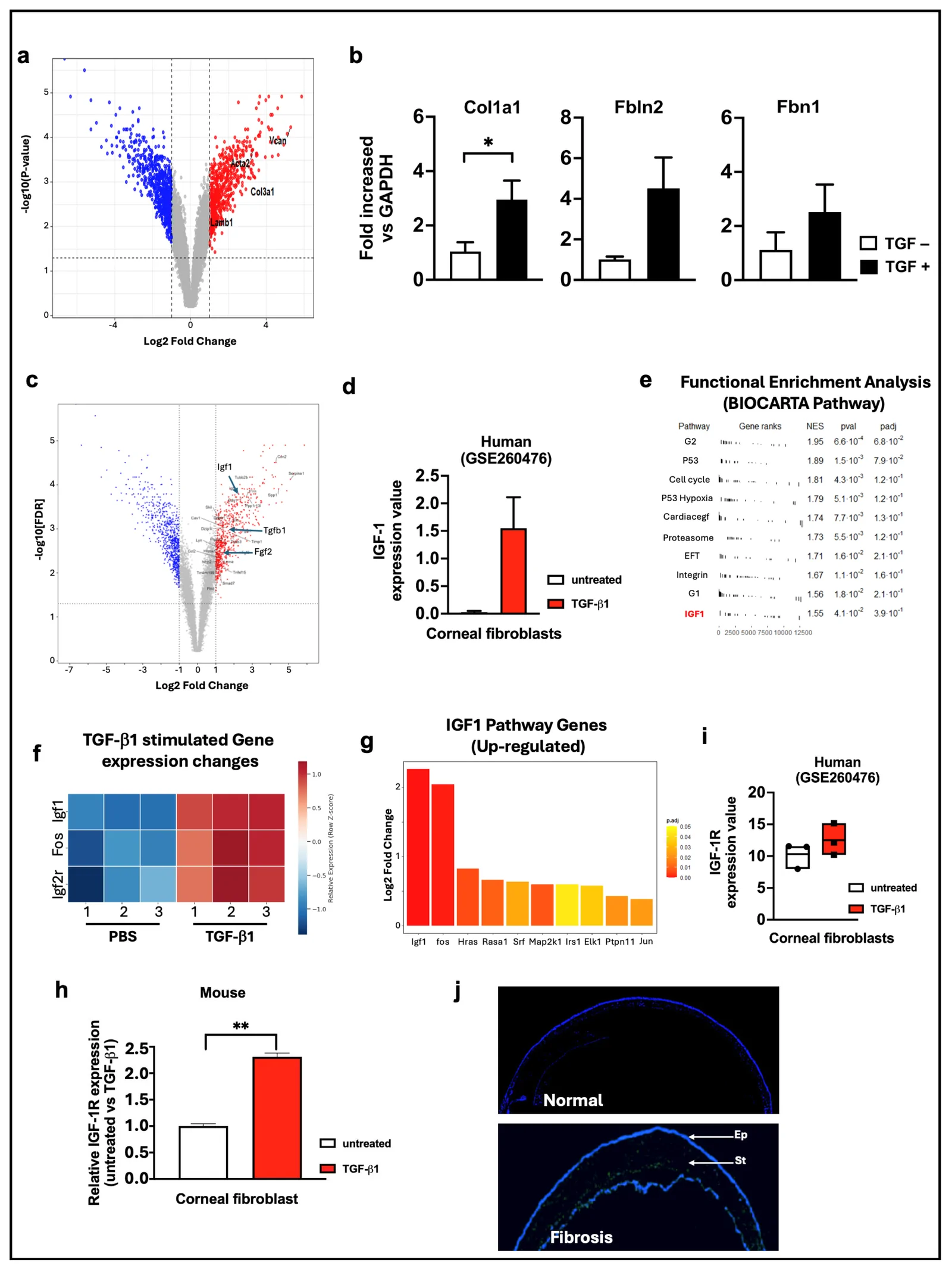
**Transcriptomic analyses identify activation of the IGF signaling pathway during corneal myofibroblast differentiation.** (**a**) Volcano plot of bulk RNA sequencing comparing primary mouse corneal fibroblasts treated with TGF-β1 (10 ng/mL, 3 days) and untreated controls. Differentially expressed genes are displayed according to log_2_ fold change and adjusted *p*-value. Representative fibrosis-associated extracellular matrix genes, including Col1a1, Fbln2, and Fbn1, are highlighted. (**b**) Quantitative RT-PCR validation of fibrosis-associated extracellular matrix genes (Col1a1, Fbln2, and Fbn1) in primary mouse corneal fibroblasts following TGF-β1 stimulation. (**c**) Volcano plot highlighting differential expression of Igf1 and other representative genes following TGF-β1-induced myofibroblast differentiation. (**d**) Validation of IGF1 expression using an independent human corneal fibroblast RNA-sequencing dataset (GSE260476). (**e**) Gene Set Enrichment Analysis (GSEA) demonstrating enrichment of signaling pathways following TGF-β1 treatment. The BIOCARTA_IGF1_PATHWAY was among the significantly enriched pathways. (**f**) Heatmap showing expression changes in representative IGF signaling pathway-related genes in untreated and TGF-β1-treated mouse corneal fibroblasts. (**g**) Representative upregulated genes within the IGF signaling pathway identified by GSEA. Bar height represents log_2_ fold change, and color indicates adjusted *p* value. (**h**) Quantitative RT-PCR validation of Igf1r expression in primary mouse corneal fibroblasts following TGF-β1 stimulation. (**i**) Validation of IGF1R expression using an independent human corneal fibroblast RNA-sequencing dataset (GSE260476). (**j**) Representative immunofluorescence staining of IGF-1R in normal and fibrotic mouse corneas. IGF-1R expression (green) was increased within the injured corneal stroma of fibrotic corneas compared with normal corneas. Nuclei were counterstained with DAPI (blue). Ep, corneal epithelium; St, corneal stroma. Data are presented as mean ± SEM. Statistical significance was determined using an unpaired two-tailed Student’s *t*-test (**b**,**h**) or as indicated for the publicly available RNA-sequencing dataset (**d**,**i**). * *p* < 0.05, ** *p* < 0.01.

Differential expression analysis identified 1390 high-confidence differentially expressed genes (DEGs), including 678 upregulated and 712 downregulated genes (FDR < 0.05; |log_2_ fold change| ≥ 1.0). A volcano plot illustrating the global transcriptional changes is shown in Figure 2a, and the complete distribution of significantly regulated genes is provided in Supplementary Figure S3. Consistent with myofibroblast differentiation, several extracellular matrix-associated genes, including Col1a1, Fbln2, and Fbn1, were among the significantly upregulated transcripts. Quantitative RT-PCR confirmed increased expression of these fibrosis-associated genes following TGF-β1 stimulation (Figure 2b).

To determine whether the IGF signaling pathway was altered during myofibroblast differentiation, we examined the RNA-seq dataset for IGF-related genes. Igf1 expression was significantly increased following TGF-β1 stimulation (Figure 2c). Gene Set Enrichment Analysis (GSEA) further demonstrated significant enrichment of the BIOCARTA_IGF1_PATHWAY, indicating coordinated activation of the IGF signaling network during myofibroblast differentiation (Figure 2e). A heatmap of representative IGF signaling-related genes demonstrated consistent upregulation across all biological replicates (Figure 2f). Representative genes contributing to this enrichment included Igf1, Fos, Hras, Rasa1, Srf, Map2k1, Irs1, Elk1, Ptpn11, and Jun (Figure 2g).

To validate these transcriptomic findings, Igf1r expression was examined by quantitative RT-PCR. TGF-β1 treatment significantly increased Igf1r mRNA expression in primary mouse corneal fibroblasts by approximately 2.3-fold compared with untreated controls (Figure 2h). Analysis of an independent human corneal fibroblast RNA-sequencing dataset (GSE260476) similarly demonstrated increased expression of both IGF1 and IGF1R following TGF-β1 stimulation (Figure 2d,i), indicating that activation of the IGF signaling pathway is conserved between mouse and human corneal fibroblasts.

To determine whether increased IGF-1R expression also occurred during corneal fibrosis in vivo, immunofluorescence staining was performed on normal and fibrotic mouse corneas. Minimal IGF-1R immunoreactivity was detected in normal corneal stroma, whereas fibrotic corneas exhibited marked stromal IGF-1R expression (Figure 2j), supporting activation of the IGF signaling axis during corneal fibrosis.

Collectively, these transcriptomic, molecular, and histological analyses demonstrate that TGF-β1-induced myofibroblast differentiation is associated with activation of the IGF signaling pathway, providing a molecular basis for subsequent functional studies investigating the role of IGF signaling in corneal myofibroblast survival.

### 2.3. IGF Signaling Promotes Corneal Myofibroblast Survival and Suppresses Apoptosis

**Figure 3:**
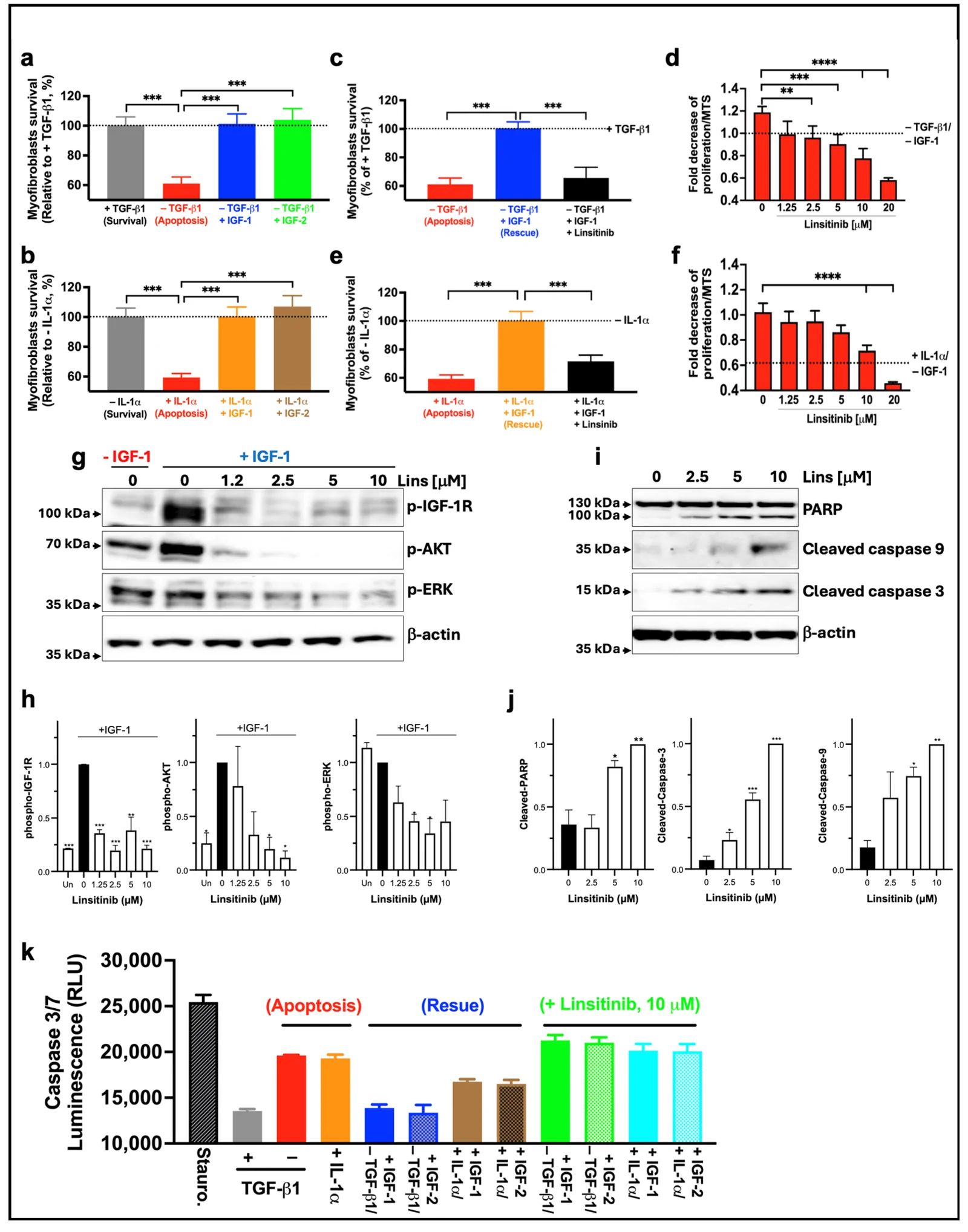
**IGF signaling promotes corneal myofibroblast survival by activating IGF-1R/AKT/ERK signaling and suppressing caspase-dependent apoptosis.** (**a**) Corneal myofibroblast survival following TGF-β1 withdrawal. Removal of TGF-β1 reduced myofibroblast survival, whereas treatment with IGF-1 or IGF-2 (100 ng/mL) restored cell survival. Cell viability was normalized to the continuous TGF-β1-treated control. (**b**) Corneal myofibroblast survival following IL-1α-induced apoptosis. IGF-1 or IGF-2 restored myofibroblast survival following IL-1α treatment. Cell viability was normalized to untreated control levels. (**c**) Effect of linsitinib (10 μM) on IGF-1-mediated rescue of myofibroblast survival following TGF-β1 withdrawal. (**d**) Dose-dependent inhibition of IGF-1-mediated myofibroblast survival by linsitinib under TGF-β1 withdrawal conditions. (**e**) Effect of linsitinib (10 μM) on IGF-1-mediated rescue of myofibroblast survival following IL-1α treatment. (**f**) Dose-dependent inhibition of IGF-1-mediated myofibroblast survival by linsitinib under IL-1α-induced apoptotic conditions. Cell survival in panels (*a*–*f*) was assessed using the MTS assay. (**g**) Representative immunoblots showing phosphorylation of IGF-1R, AKT, and ERK following IGF-1 stimulation in the presence of increasing concentrations of linsitinib. β-Actin served as the loading control. (**h**) Densitometric quantification of phosphorylated IGF-1R, AKT, and ERK from three independent Western blot experiments. (**i**) Representative immunoblots showing PARP, cleaved caspase-9, and cleaved caspase-3 following linsitinib treatment. β-Actin served as the loading control. (**j**) Densitometric quantification of cleaved PARP, cleaved caspase-9, and cleaved caspase-3 from three independent Western blot experiments. (**k**) Caspase-3/7 activity assay demonstrating apoptosis under TGF-β1 withdrawal and IL-1α treatment and rescue by IGF-1 or IGF-2. Linsitinib (10 μM) restored caspase-3/7 activity despite IGF treatment. Staurosporin served as the positive control for apoptosis. Data are presented as mean ± SEM from six independent biological experiments for the MTS assays (**a**–**f**), three independent biological experiments for Western blot quantification (**h**,**j**), and three independent biological experiments for the Caspase-3/7 activity assay (**k**). Statistical significance was determined using one-way ANOVA followed by Tukey’s multiple-comparison test. * *p* < 0.05, ** *p* < 0.01, *** *p* < 0.001, **** *p* < 0.0001. Dotted lines indicate the reference levels of the corresponding control groups (**a**–**f**).

To determine whether IGF signaling regulates corneal myofibroblast survival, differentiated myofibroblasts were subjected to two established pro-apoptotic conditions, withdrawal of TGF-β1 and exposure to IL-1α. Consistent with previous studies, removal of TGF-β1 significantly reduced myofibroblast survival, decreasing cell viability to approximately 60% of control levels (Figure 3a). Addition of either IGF-1 or IGF-2 (each 100 ng/mL) restored myofibroblast survival to levels comparable to those maintained in the presence of TGF-β1. IL-1α treatment significantly reduced myofibroblast survival, whereas co-treatment with either IGF-1 or IGF-2 restored cell survival to near-baseline levels (*n* = 6) (Figure 3b). Together, these findings indicate that both IGF-1 and IGF-2 protect corneal myofibroblasts from apoptosis induced by two distinct pro-apoptotic stimuli.

To determine whether these pro-survival effects were mediated through IGF-1R signaling, differentiated myofibroblasts were treated with linsitinib, an inhibitor of both IGF-1R and INSR. Pharmacologic inhibition of IGF signaling abolished the protective effect of IGF-1 following both TGF-β1 withdrawal and IL-1α exposure (Figure 3c,e). Linsitinib reduced myofibroblast survival in a dose-dependent manner under both apoptotic conditions (Figure 3d,f). Comparable dose-dependent inhibition was also observed in IGF-2 treated myofibroblasts subjected to TGF-β1 withdrawal and IL-1α exposure (Supplementary Figure S4), demonstrating that the survival-promoting effects of both IGF ligands require IGF-1R/INSR signaling.

To investigate the downstream signaling mechanisms responsible for IGF-mediated survival, corneal myofibroblasts were stimulated with IGF-1 (100 ng/mL) in the presence or absence of increasing concentrations of linsitinib for 4 h. IGF-1 markedly increased phosphorylation of IGF-1R, AKT, and ERK, whereas linsitinib suppressed phosphorylation of all three molecules in a dose-dependent manner (Figure 3g,h), indicating effective inhibition of canonical IGF signaling.

We next examined whether inhibition of IGF signaling activates apoptotic pathways. Treatment with linsitinib for 24 h induced dose-dependent cleavage of PARP, caspase-9, and caspase-3, consistent with activation of the intrinsic apoptotic pathway (Figure 3i,j). To directly quantify apoptosis, Caspase-3/7 activity assays were performed under both TGF-β1 withdrawal and IL-1α-induced conditions. Withdrawal of TGF-β1 or IL-1α treatment significantly increased caspase-3/7 activity, whereas both IGF-1 and IGF-2 suppressed caspase activation (*n* = 3). Importantly, linsitinib restored caspase-3/7 activity despite IGF treatment, demonstrating that pharmacological inhibition of IGF signaling reverses the anti-apoptotic effects of IGF ligands (Figure 3k).

### 2.4. Inhibition of IGF-1R/INSR Signaling Reduces Corneal Fibrosis and Myofibroblast Persistence In Vivo

**Figure 4:**
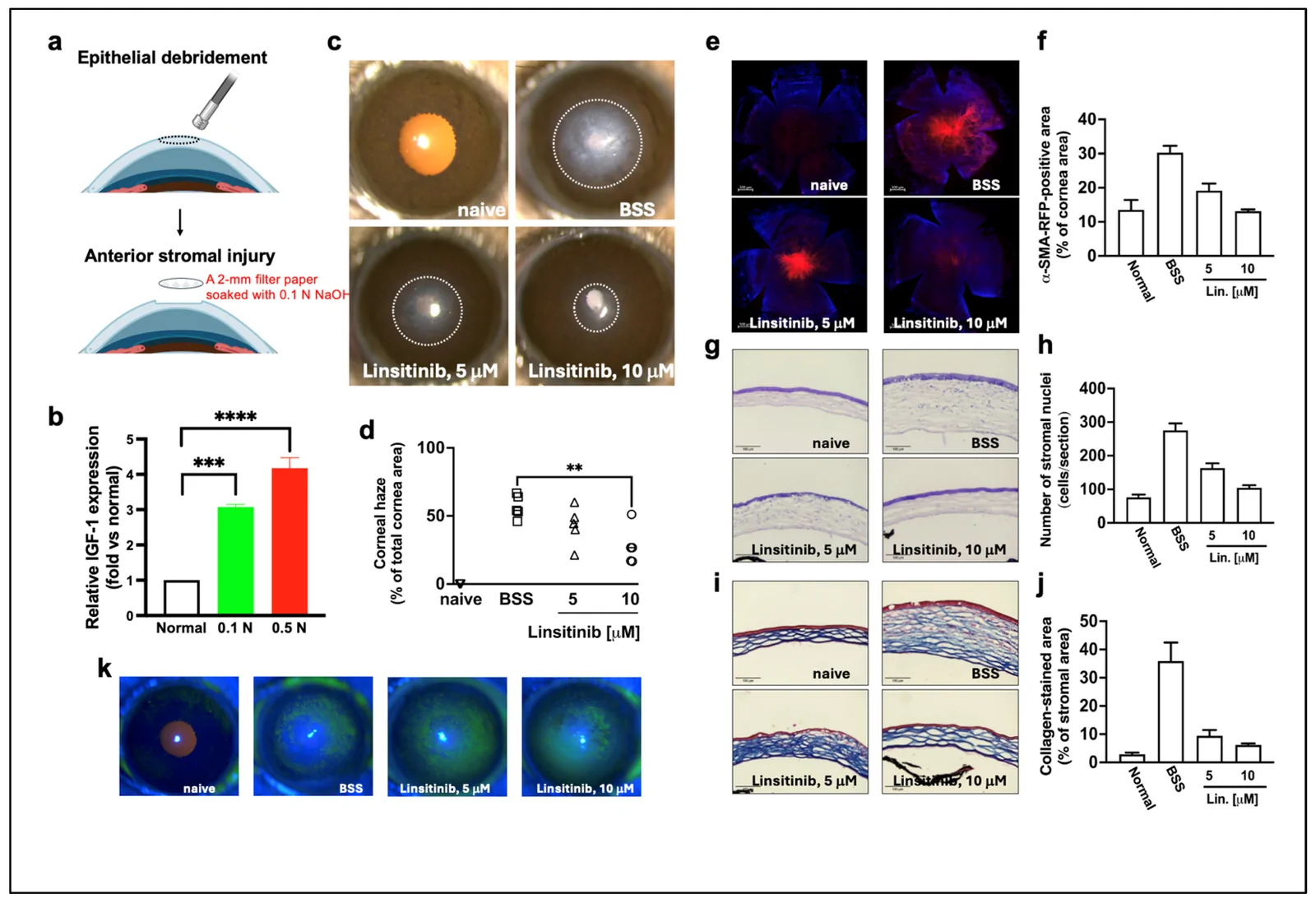
**Pharmacological inhibition of IGF-1R/INSR signaling attenuates corneal fibrosis, reduces myofibroblast accumulation, and preserves epithelial wound healing in vivo.** (**a**) Schematic illustration of the murine corneal fibrosis model. Central corneal epithelial debridement was followed by localized anterior stromal injury using a 2-mm filter paper soaked in 0.1 N NaOH. (**b**) Quantitative RT-PCR analysis of Igf1 expression following alkali injury. Expression levels were normalized to uninjured corneas. (**c**) Representative slit-lamp images of corneas 14 days after injury following subconjunctival administration of vehicle (BSS) or linsitinib. (**d**) Quantification of the corneal haze area from slit-lamp images (*n* = 5). (**e**) Representative whole-mount images of Tg(α-SMA-RFP) corneas showing α-SMA-positive myofibroblasts (red). Nuclei were counterstained with DAPI (blue) (*n* = 2). (**f**) Quantification of α-SMA-RFP-positive area expressed as a percentage of the total corneal area. (**g**) Representative hematoxylin and eosin (H&E)-stained corneal sections collected 14 days after injury (*n* = 2). (**h**) Quantification of stromal cellularity from H&E-stained sections. (**i**) Representative Masson’s trichrome-stained corneal sections showing stromal collagen organization (*n* = 2). (**j**) Quantification of trichrome-positive area expressed as a percentage of the stromal area. (**k**) Representative fluorescein staining images demonstrating corneal epithelial wound closure 14 days after injury. Data are presented as mean ± SEM. Quantitative analyses of α-SMA-RFP-positive area expressed (**f**), H&E (**h**) and Masson’s trichrome staining (**j**) are presented descriptively, the consequence of limited biological sample numbers. Statistical significance was determined using one-way ANOVA followed by Tukey’s multiple-comparison test. ** *p* < 0.01, *** *p* < 0.001, **** *p* < 0.0001.

To investigate the role of IGF signaling during corneal fibrosis, a murine model of stromal fibrosis was established by combining central epithelial debridement with localized anterior stromal injury induced by 0.1 N NaOH (Figure 4a). We first examined whether IGF signaling was associated with the corneal injury response. Quantitative RT-PCR demonstrated increased *Igf1* expression 5 days after injury, with greater induction following 0.5 N NaOH injury than following 0.1 N NaOH injury (*n* = 3) (Figure 4b), indicating an injury severity-dependent increase in *Igf1* expression.

To determine whether pharmacological inhibition of IGF-1R/INSR signaling attenuates corneal fibrosis, Tg(α-SMA-RFP) mice received subconjunctival administration of vehicle (BSS) or linsitinib following injury. Slit-lamp examination on day 14 demonstrated prominent stromal haze in vehicle-treated corneas, whereas linsitinib treatment reduced corneal opacity in a dose-dependent manner (*n* = 5) (Figure 4c). Quantification of haze area confirmed a significant reduction following linsitinib treatment, with the greatest reduction observed at 10 μM (*n* = 5 mice per group; Figure 4d).

We next evaluated α-SMA-RFP-positive myofibroblasts within the injured corneas. Vehicle-treated corneas exhibited extensive α-SMA-RFP fluorescence within the central stromal region, whereas treatment with linsitinib reduced the α-SMA-RFP-positive area (Figure 4e). Quantitative analysis demonstrated a dose-dependent reduction in α-SMA-RFP-positive area relative to the total corneal area following linsitinib treatment (Figure 4f).

Histological analysis further demonstrated changes in stromal cellularity and architecture. H&E-stained sections from vehicle-treated corneas exhibited increased stromal cellularity and disruption of normal stromal organization compared with naïve corneas, whereas linsitinib-treated corneas exhibited reduced stromal cellularity and normalization of stromal architecture (Figure 4g). Quantification of stromal nuclei showed a reduction in stromal cellularity following linsitinib treatment (Figure 4h).

Masson’s trichrome staining was performed to assess fibrotic stromal remodeling. Vehicle-treated corneas exhibited increased trichrome-positive stromal area and disrupted collagen organization, whereas linsitinib-treated corneas showed reduced collagen accumulation and restoration of a more organized stromal extracellular matrix (Figure 4i). Quantitative analysis demonstrated a reduction in trichrome-positive area relative to the total stromal area following linsitinib treatment (Figure 4j).

Finally, fluorescein staining was performed to assess corneal epithelial integrity on day 14. No residual epithelial defects were evident in vehicle- or linsitinib-treated corneas, indicating that local inhibition of IGF-1R/INSR signaling did not impair epithelial wound closure under these experimental conditions (Figure 4k).

Collectively, these in vivo findings demonstrate that pharmacological inhibition of IGF-1R/INSR signaling reduces corneal haze, α-SMA-positive myofibroblast accumulation, stromal cellularity, and fibrotic extracellular matrix remodeling without compromising epithelial wound closure.

## 3. Discussion

**Figure 5:**
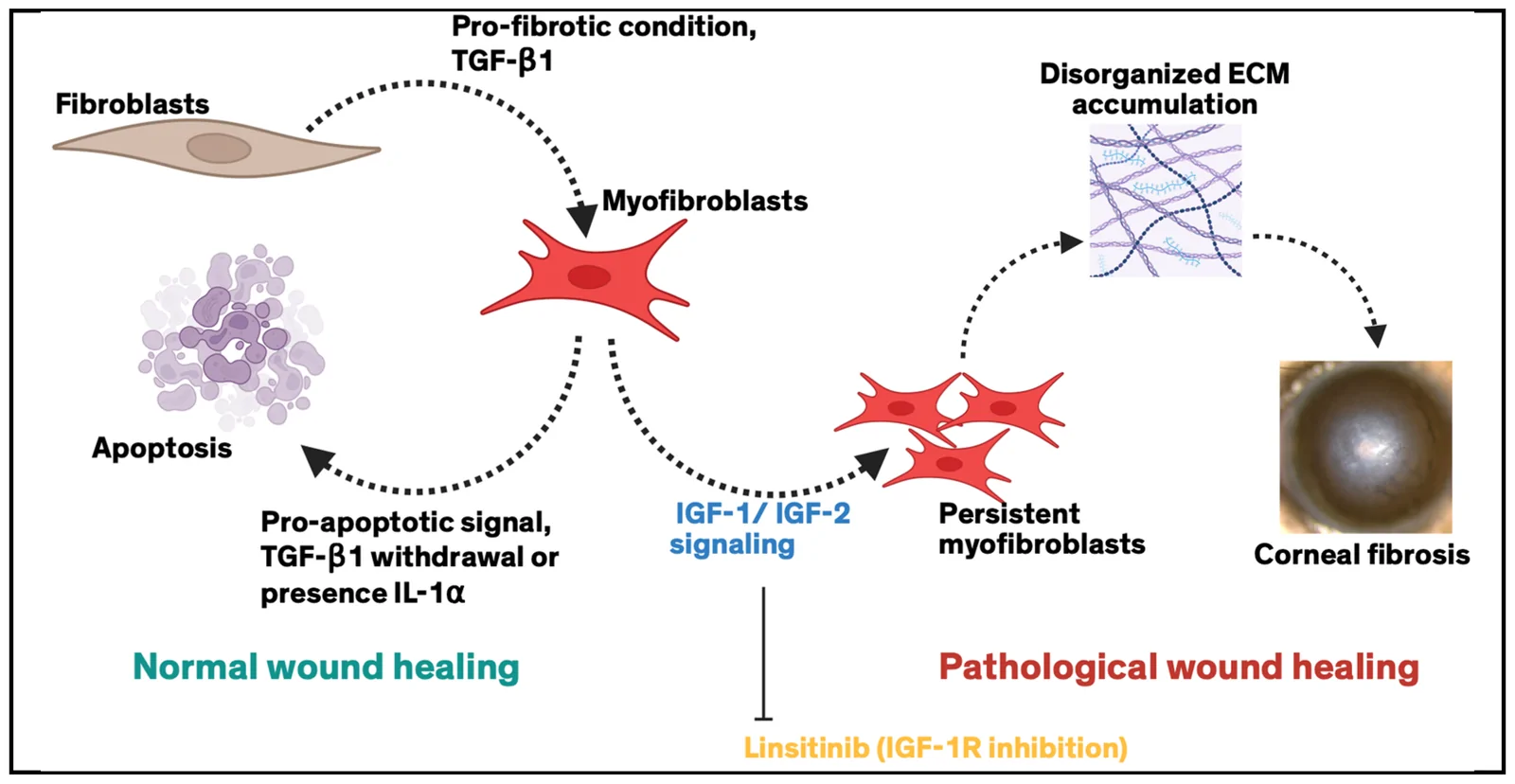
**Proposed model illustrating the role of IGF signaling in corneal myofibroblast persistence and fibrosis.** During normal corneal wound healing, fibroblasts differentiate into myofibroblasts in response to profibrotic stimuli, such as transforming growth factor-β1 (TGF-β1), to facilitate tissue repair. Following restoration of tissue homeostasis, withdrawal of profibrotic signals and exposure to pro-apoptotic mediators, including interleukin-1 (IL-1), promote myofibroblast apoptosis, thereby limiting extracellular matrix (ECM) deposition and preserving corneal transparency. Under pathological conditions, survival factors such as insulin-like growth factors (IGFs) activate IGF-1 receptor (IGF-1R)/insulin receptor (INSR) signaling, promoting myofibroblast survival and persistence. Persistent myofibroblasts produce excessive and disorganized ECM, leading to stromal fibrosis and loss of corneal transparency. Pharmacological inhibition of IGF-1R/INSR signaling with linsitinib restores myofibroblast apoptosis, reduces myofibroblast persistence, and attenuates corneal fibrosis.

Corneal fibrosis remains a major cause of vision loss worldwide, yet the mechanisms that sustain myofibroblast persistence after differentiation remain poorly understood. The present study identified IGF signaling as a previously unrecognized regulator of corneal myofibroblast survival. Rather than promoting myofibroblast differentiation, our findings support a model in which IGF signaling maintains the persistence of differentiated myofibroblasts, thereby contributing to chronic fibrotic remodeling. These findings shift attention from mechanisms that generate myofibroblasts to those that prevent their elimination and identify survival signaling as a potential therapeutic target in corneal fibrosis (Figure 5).

An important conceptual advance of this study is the distinction between differentiation and persistence. TGF-β signaling has long been recognized as the principal driver of myofibroblast differentiation, whereas considerably less attention has been directed toward the mechanisms that sustain differentiated myofibroblasts. Our findings support the idea that IGF signaling primarily functions as a survival pathway rather than a differentiation signal, suggesting that persistent fibrosis depends not only on continued myofibroblast generation but also on failure of myofibroblast disposal.

The increased responsiveness of differentiated myofibroblasts to IGF signaling is consistent with the concept that mature myofibroblasts become increasingly dependent on survival cues within the injured microenvironment. Consistent with our findings, IGF signaling has been implicated in promoting myofibroblast survival and persistence in several fibrotic tissues. In pulmonary fibrosis, macrophage-derived IGF-I protects myofibroblasts from apoptosis through activation of Akt and ERK signaling, thereby contributing to persistent fibroblast accumulation [39]. Similar profibrotic roles for IGF signaling have also been reported in hepatic and orbital fibrosis [40,41,42].

The ability of IGF signaling to counteract diverse pro-apoptotic stimuli suggests that it functions as a central survival pathway rather than merely modifying a single apoptotic trigger. Activation of AKT and ERK downstream of IGF-1R is well established as a mechanism promoting cell survival in multiple tissues [43,44]. Our findings extend these pathways contributes to the pathological longevity of corneal myofibroblasts.

Components of the IGF axis are likewise expressed in the cornea, where they contribute to epithelial homeostasis and wound healing [45]. Previous studies have demonstrated expression of IGF ligands and IGF-1R in corneal epithelial and stromal cells, supporting the existence of a local IGF signaling network. The increased Igf1 expression observed following corneal injury is therefore consistent with activation of local IGF signaling during fibrotic wound healing. However, because the present study measured whole-cornea Igf1 expression, the cellular sources of IGF-1 and IGF-2 and the relative contributions of locally produced versus circulating IGFs to myofibroblast persistence remain to be determined. Nevertheless, regardless of the source of IGFs, our findings demonstrate that activation of the IGF-1R/INSR signaling axis is required for corneal myofibroblast survival and that pharmacological inhibition of this pathway reduces fibrosis in vivo.

Several limitations of this study should be considered. First, although the in vivo experiments consistently demonstrated significant antifibrotic effects of linsitinib across multiple independent outcome measures, the relatively modest sample size warrants validation in larger preclinical studies. Second, linsitinib inhibits both IGF-1R and INSR and therefore does not distinguish the relative contributions of IGF-1R, INSR, and hybrid IGF-1R/INSR to corneal myofibroblast persistence. Although our findings establish the importance of the IGF signaling axis in regulating corneal fibrosis, future studies employing receptor-specific knockdown, selective pharmacological inhibitors, or conditional genetic models will be required to define the individual roles of these receptor complexes. Third, although AKT and ERK were identified as major downstream mediators of IGF signaling, additional pathways may also contribute to myofibroblast survival and should be investigated. Finally, the cellular sources of IGF ligands within the injured corneal microenvironment remain incompletely defined. Corneal epithelial cells, stromal fibroblasts, resident keratocytes, and infiltrating immune cells, such as fibrocytes, may all contribute to local IGF production through autocrine and paracrine signaling. Future studies using cell type-specific genetic approaches and spatial transcriptomics will help define the cellular circuitry regulating IGF-dependent myofibroblast persistence. From a translational perspective, local subconjunctival delivery of linsitinib effectively reduced stromal fibrosis without impairing epithelial wound healing in the present study. Nevertheless, further studies comparing local and systemic delivery strategies, together with long-term safety evaluations, will be necessary to define the optimal therapeutic window while preserving corneal tissue homeostasis.

In conclusion, this study identifies the IGF axis as a critical regulator of corneal myofibroblast survival. Unlike TGF-β, which primarily drives myofibroblast differentiation, IGF-1 and IGF-2 promote persistence of differentiated myofibroblasts by activating survival signaling pathways and suppressing apoptosis. Pharmacologic inhibition of IGF-1R/INSR signaling restores apoptotic responses and attenuates corneal fibrosis in vivo. These findings establish IGF-dependent survival signaling as a previously unrecognized mechanism contributing to corneal fibrosis and highlight myofibroblast survival pathways as potential therapeutic targets for fibrotic disease.

## 4. Materials and Methods

### 4.1. Materials

Linsitinib (OSI-906) was purchased from MedChemExpress LLC, (Monmouth Junction, NJ, USA). A 20 mM stock solution was prepared in dimethyl sulfoxide (DMSO) and stored at −20 °C. For in vitro experiments, the stock solution was diluted in Dulbecco’s Modified Eagle Medium (DMEM) to the indicated concentrations. For in vivo studies, linsitinib was diluted in balanced salt solution (BSS) immediately before administration. Recombinant mouse transforming growth factor-β1 (TGF-β1), recombinant mouse interleukin-1α (IL-1α) recombinant mouse IGF-1 and recombinant mouse IGF-2 were purchased from R&D Systems (Minneapolis, MN, USA) and reconstituted according to the manufacturer’s instructions to generate a stock solution of 50 μg/mL. Staurosporin was purchased from Sigma-Aldrich (St. Louis, MO, USA).

Alexa Fluor 647-conjugated anti-α-smooth muscle actin (α-SMA) antibody was purchased from Invitrogen (Waltham, MA, USA). Dulbecco’s Modified Eagle Medium, phosphate-buffered saline (PBS), fetal bovine serum (FBS), and other routine cell culture reagents were obtained from Sigma-Aldrich unless otherwise specified.

### 4.2. Isolation and Culture of Mouse Corneal Epithelial Cells and Fibroblasts

Primary mouse corneal epithelial cells and corneal fibroblasts were isolated from 16 corneas obtained from 8 C57BL/6J mice with minor modifications. Briefly, whole corneas were excised under a dissecting microscope. For epithelial cell isolation, corneas were cut into four quadrants including the limbus and placed epithelial side down on 0.1% gelatin-coated 12-well culture plates. Corneal explants were initially cultured in DMEM/F12 (Sigma-Aldrich), and complete corneal epithelial culture medium was gradually added on day 2. After approximately 7 days, the corneal explants were removed, and the epithelial cells were maintained in fresh corneal epithelial culture medium. The culture medium was replaced every 3–4 days until the cells reached confluence.

For corneal fibroblast isolation, freshly isolated corneas were incubated in 500 μL dispase II solution (Gibco, Waltham, MA, USA) at 4 °C overnight. The corneal epithelial sheet and endothelial cells were removed, and the remaining stromal tissues were washed twice with 1% antibiotic–antimycotic in PBS. Stromal tissues were then digested in 500 μL of 1 mg/mL collagenase A (Roche, Mannheim, Germany) at 37 °C for 1–2 h with gentle pipetting every 10 min. Dissociated cells were filtered through a 40 μm cell strainer, centrifuged at 1000× *g* for 5 min, and resuspended in corneal stromal fibroblast culture medium. Cells were cultured in 6-well plates at 37 °C in a humidified incubator containing 5% CO_2_, and the culture medium was replaced every 2–3 days until confluence. Corneal fibroblasts between passages 1 and 3 were used for all experiments.

### 4.3. MTS Assay and Caspase 3/7 Assay

Cellular metabolic activity was assessed using the CellTiter 96^®^ AQueous One Solution Cell Proliferation Assay (MTS assay; Promega, Madison, WI, USA) according to the manufacturer’s instructions. Primary mouse corneal fibroblasts were seeded at 1 × 10^4^ cells per well in 96-well plates and cultured overnight in complete growth medium. For differentiation experiments, cells were treated with TGF-β1 (10 ng/mL) for 3 days to induce myofibroblast differentiation. To evaluate cell survival under pro-apoptotic conditions, differentiated myofibroblasts were washed with pre-warmed PBS and cultured in serum-free DMEM or DMEM containing IL-1α, as indicated. For experiments involving IGF signaling inhibition, linsitinib was added at the indicated concentrations at the time of treatment. The concentration of IGF-1 and IGF-2 (100 ng/mL) was selected based on preliminary concentration-response experiments (1, 10, and 100 ng/mL), in which 100 ng/mL produced the greatest increase in corneal fibroblast proliferation without inducing myofibroblast differentiation. At the designated time points, 10 μL of MTS reagent was added to each well and incubated for 4 h at 37 °C. Absorbance was measured using a microplate reader at 490 nm. Cellular metabolic activity was expressed relative to the appropriate control group. Six technical replicate wells were included for each experimental condition, and all experiments were independently repeated at least three times. To directly assess apoptosis, caspase-3/7 activity was measured under the same pro-apoptotic conditions using a luminescence-based Caspase-Glo^®^ 3/7 Assay (Promega, Madison, WI, USA) according to the manufacturer’s instructions. Following TGF-β1 withdrawal or IL-1α exposure, cells were treated with IGF-1 or IGF-2 (100 ng/mL) in the presence or absence of linsitinib (10 μM). Caspase-Glo^®^ 3/7 reagent was added to each well, and luminescence was measured using a microplate reader. Staurosporin-treated cells were included as a positive control for apoptosis. Caspase-3/7 activity was expressed as relative luminescence units (RLU). Independent biological experiments were performed as indicated in the corresponding figure legend.

### 4.4. Bulk RNA Sequencing and Quantitative PCR Analysis

Primary mouse corneal fibroblasts were treated with transforming growth factor-β1 (TGF-β1; 10 ng/mL) for 3 days to induce myofibroblast differentiation, whereas control cells received vehicle (PBS) treatment. Total RNA was isolated using the RNeasy Mini Kit (Qiagen, Hilden, Germany) according to the manufacturer’s instructions. RNA concentration and integrity were assessed prior to library preparation.

RNA sequencing (RNA-seq) libraries were prepared and sequenced by Novogene (Sacramento, CA, USA). RNA-seq analysis was performed using three independent biological replicates per group (*n* = 3). Sequencing generated between 38.9 and 43.2 million reads per mate (~77.8–86.4 million total reads per sample). Sequence quality was assessed using MultiQC, with mean Phred quality scores of approximately 35 across all read positions. Low-expressed genes (raw counts < 10) and transcripts with duplicate gene assignments were removed prior to analysis. Count data were normalized using the Trimmed Mean of M-values (TMM) method and log_2_-transformed to stabilize variance. Differential expression was evaluated using an Integrative Hypothesis Testing approach that combined Student’s *t*-test and Median Difference Test statistics using Stouffer’s method. Multiple testing correction was performed using the Benjamini–Hochberg false discovery rate (FDR) procedure. Genes with FDR < 0.05 and |log_2_ fold change| ≥ 1.0 were considered significantly differentially expressed. Gene Set Enrichment Analysis (GSEA 4.4.0) was subsequently performed using the Molecular Signatures Database (MSigDB v5.2, Broad Institute, Cambridge, MA, USA), and enriched pathways were evaluated using the normalized enrichment score (NES) and FDR-adjusted *p* values.

For quantitative real-time PCR (qPCR), 1 μg of total RNA was reverse transcribed using the iScript™ cDNA Synthesis Kit (Bio-Rad Laboratories, Hercules, CA, USA) according to the manufacturer’s instructions. Quantitative PCR was performed using iTaq™ Universal SYBR^®^ Green Supermix (Bio-Rad Laboratories) on a QuantStudio™ 7 Flex Real-Time PCR System (Applied Biosystems, Thermo Fisher Scientific, Waltham, MA, USA). Relative gene expression was calculated using the 2^−ΔΔCt method and normalized to Gapdh as the endogenous reference gene. All primer sequences used in this study are provided in Supplementary Table S1.

### 4.5. Western Blots

For signaling studies, corneal myofibroblasts were serum-starved overnight and stimulated with recombinant mouse IGF-1 (100 ng/mL) in the presence of linsitinib (0–10 μM) for 4 h. Cells were lysed in RIPA buffer (Thermo Fisher Scientific, Waltham, MA, USA) supplemented with protease and phosphatase inhibitor cocktails. Total protein concentrations were determined using the Bradford protein assay (Bio-Rad, Hercules, CA, USA). Equal amounts of protein (20 μg per sample) were separated on NuPAGE 10% Bis-Tris gel (Invitrogen, Carlsbad, CA, USA) and transferred onto nitrocellulose membranes. Membranes were blocked with 3% non-fat dry milk in Tris-buffered saline containing 0.1% Tween-20 (TBS-T) for 1 h at room temperature and incubated overnight at 4 °C with primary antibodies diluted 1:1000 in blocking buffer. Antibodies against phospho-IGF-1R (Tyr1131, #3021), phospho-Akt (Ser 473, #9271), phospho-ERK1/2 (Thr202/Tyr204, #9101), PARP (#9542), cleaved-caspase-3 (#9661) and cleaved-caspase-9 (#9505) were obtained from Cell Signaling Technology (Danvers, MA, USA). After washing in Tris-buffered saline containing 0.1% Tween 20 (TBS-T), membranes were then incubated for 1 h with horseradish peroxidase (HRP)-conjugated goat anti-rabbit or goat anti-mouse antibodies (Cell Signaling Technology; 1: 5000) as the secondary antibody. The membranes were developed using iBright 1500 Imager (Thermo Fisher Scientific, Waltham, MA, USA) and enhanced chemiluminescence detection (Amersham Bioscience, Buckinghamshire, UK). β-actin was used as a loading control.

### 4.6. Murine Model of Corneal Fibrosis

Wild-type C57BL/6J and Tg(α-SMA-RFP) mice were obtained from The Jackson Laboratory (Bar Harbor, ME, USA) and maintained in the University of Illinois Chicago (UIC) animal facility under specific pathogen-free conditions. Male and female mice aged 8–10 weeks were used for all experiments. All animal procedures were approved by the Institutional Animal Care and Use Committee (IACUC) of the University of Illinois Chicago and were conducted in accordance with the ARVO Statement for the Use of Animals in Ophthalmic and Vision Research. Tg(α-SMA-RFP) mice were genotyped before use in subsequent in vivo corneal fibrosis experiments. Genomic DNA was extracted from tail tissue using the Direct Mouse Genotyping Kit (APExBIO, Houston, TX, USA) according to the manufacturer’s instructions. Briefly, tail tissue samples were lysed using the lysis buffer supplemented with Proteinase K for 15 min at 56 °C, followed by heat inactivation for 20 min at 95 °C. Balance buffer was then added, and the resulting lysate was used directly as the PCR template with the kit’s 2× PCR Master Mix. PCR amplification was performed using RFP-specific primers (forward, 5′-GCCAAGCTGAAGGTGACCAA-3′; reverse, 5′-TTGGAGCCGTACTGGAACTG-3′), with the following cycling conditions: initial denaturation at 98 °C for 1 min; 30 cycles of denaturation at 98 °C for 15 s, annealing at 58 °C for 15 s, and extension at 72 °C for 30 s; followed by a final extension at 72 °C for 2 min. PCR products were resolved by 2% agarose gel electrophoresis and visualized under UV illumination to confirm the expected amplicon.

Mice were anesthetized by intraperitoneal injection of ketamine and xylazine, and adequate anesthesia was confirmed by the absence of a toe-pinch reflex. Topical proparacaine hydrochloride ophthalmic solution (0.5%) was applied for local anesthesia. A 2-mm central corneal region was demarcated using a biopsy punch, and the corneal epithelium was removed using an AlgerBrush II corneal rust ring remover (Alger Company, Lago Vista, TX, USA). To induce corneal fibrosis, anterior stromal injury was created by applying a 2-mm diameter filter paper soaked in either 0.1 N NaOH to the central cornea for 30 s. Following alkali exposure, the ocular surface was thoroughly irrigated with 10 mL sterile saline solution to remove residual NaOH. Following corneal injury, mice were randomly assigned to treatment groups (*n* = 5 mice per group). Linsitinib or vehicle (balanced salt solution, BSS, 5 μL) was administered by subconjunctival injection once weekly throughout the experimental period (total twice). Epithelial defects were visualized by topical application of fluorescein sodium solution followed by saline irrigation and examination under cobalt blue illumination. Corneal opacity (haze) was evaluated by slit-lamp biomicroscopy (Intertek, London, UK) at the indicated time points. Representative slit-lamp images were acquired using identical microscope settings, including illumination intensity, magnification, exposure time, and camera settings, for all experimental groups and analyzed using ImageJ software 1.54p (National Institutes of Health, Bethesda, MD, USA).

### 4.7. Corneal Whole Mount and Histology

At the indicated time points, mice were euthanized and corneas were harvested for whole-mount imaging and histological analysis. For whole-mount imaging, eyes were excised, fixed in 4% paraformaldehyde for 10 min at room temperature, washed with phosphate-buffered saline (PBS), and the excised corneas were mounted on glass slides with the endothelial side down. Nuclei were counterstained with DAPI (Vector Laboratories, Newark, CA, USA). Fluorescence images were acquired using a LSM 980 confocal microscope with Airyscan 2 (Carl Zeiss Microscopy GmbH, Jena, Germany) under identical imaging settings for all experimental groups. α-SMA-RFP-positive areas were quantified using ImageJ software 1.54p (National Institutes of Health, Bethesda, MD, USA).

For IGF-1R immunofluorescence, frozen eye sections (10 μm) stored at −20 °C were equilibrated to room temperature, and a hydrophobic barrier was drawn around each tissue section using a Dako Pen. Sections were fixed in 10% neutral-buffered formalin for 10–15 min at room temperature, followed by incubation with Peroxidase Blocking Solution and Protein Block Solution (DAKO, Agilent Technologies, Santa Clara, CA, USA) for 10 min each at room temperature. Sections were incubated overnight at 4 °C with an anti-IGF-1R antibody (1:100; Santa Cruz Biotechnology, Dallas, TX, USA), washed three times with PBS, and incubated with fluorescein isothiocyanate (FITC)-conjugated anti-mouse IgG secondary antibody (1:200; BD Pharmingen, San Diego, CA, USA) for 1 h at room temperature. Following three washes with PBS, nuclei were counterstained with DAPI for 1 min. Sections were mounted using Fluoro-Gel mounting medium (Electron Microscopy Sciences, Hatfield, PA, USA) and imaged using a Leica Stellaris 8 confocal microscope (Leica Microsystems, Wetzlar, Germany).

For histological analysis, enucleated eyes were fixed in 10% neutral-buffered formalin, embedded in paraffin, and sectioned at 8-μm thickness. Sections were stained with hematoxylin and eosin (H&E) according to standard protocols and imaged using an Echo Revolve RVL2-K2 microscope (Echo Laboratories, San Diego, CA, USA). Histological evaluation was performed in a masked manner. All fluorescence and slit-lamp images were acquired using identical imaging settings within each experiment. Quantitative analyses were performed using predefined analysis criteria, and the same analysis workflow was applied uniformly across all experimental groups to ensure objective, consistent, and reproducible quantification.

### 4.8. Statistics

All data are presented as mean ± standard error of the mean (SEM). Statistical analyses were performed using GraphPad Prism 11 (GraphPad Software, San Diego, CA, USA). Data were obtained from independent biological replicates generated under comparable experimental conditions, and no obvious deviations from normality or homogeneity of variance were observed. Comparisons between two groups were performed using an unpaired two-tailed Student’s *t*-test. Comparisons among multiple groups were analyzed using one-way analysis of variance (ANOVA) followed by Tukey’s multiple-comparison test. A value of *p* < 0.05 was considered statistically significant.

## 5. Patents

V.K.A. and K.N.S. are inventors on a pending patent application owned by the Regents of the University of Michigan covering the use of linsitinib-mediated IGF-1R inhibition for the treatment of corneal fibrosis. K.-y.H. is inventor on a pending patent application owned by The Board of Trustees of the University of Illinois covering the use of linsitinib-mediated IGF-1R inhibition for the treatment of corneal fibrosis. T.J.S. was issued patents for targeting IGF-IR as therapy for autoimmune diseases, including TAO, held by Lundquist Institute/UCLA and University of Michigan.

## Data Availability

Restrictions apply to the availability of these data. Data were obtained from [46] and are available [https://uofi.box.com/s/2y6vts7a3kggbx0ycprzqtp36q48t6bf] (accessed on 9 August 2026) with the permission of [46].

## Supplementary Materials

The following supporting information can be downloaded at: https://www.mdpi.com/article/10.3390/ijms27167320/s1.

## Abbreviations

The following abbreviations are used in this manuscript:

IGF	Insulin-like growth factor
INSR	Insulin receptor
TGF	Transforming growth factor
IL	Interleukin
AKT	Protein kinase B
ECM	Extracellular matrix
PARP	Poly(ADP-ribose) polymerase
RFP	Red fluorescent protein
MTS	3-(4,5-Dimethylthiazol-2-yl)-5-(3-carboxymethoxyphenyl)-2-(4-sulfophenyl)-2H-tetrazolium assay
