# Supplementary material for: Insulin-like Growth Factor Signaling Promotes Corneal Myofibroblast Survival and Sustains Corneal Fibrosis
Source: Int J Mol Sci. 2026 Aug 16;27(16):7320. doi: 10.3390/ijms27167320 (PMC13513157; doi:10.3390/ijms27167320)
Supplement: Supplementary file 1 [file ijms-27-07320-s001.zip › ijms-4485451-supplementary.pdf]

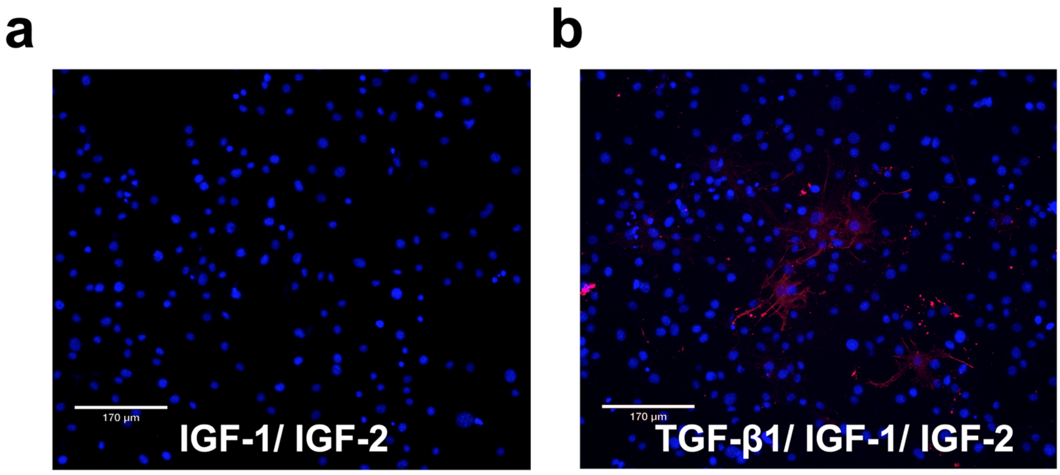

Supplementary Figure S1. IGF co-treatment does not enhance TGF-β1-induced corneal myofibroblast differentiation.
(**a**) Representative immunofluorescence images of α-smooth muscle actin (α-SMA) expression in primary mouse corneal fibroblasts treated with IGF-1 (100 ng/mL), IGF-2 (100 ng/mL), or combined IGF-1 and IGF-2 (100 ng/mL each). (**b**) Representative immunofluorescence images of α-SMA expression in corneal fibroblasts treated with TGF-β1 (10 ng/mL) alone or in combination with IGF-1 and IGF-2. Nuclei were counterstained with DAPI (blue), and α-SMA is shown in red. Scale bar = 170 µm.

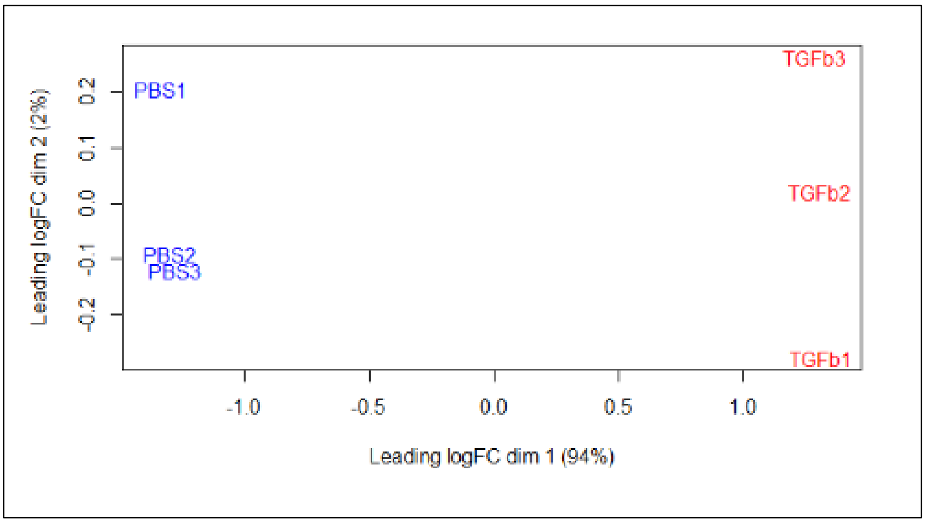

Supplementary Figure S2. Multidimensional Scaling (MDS) Plot. Sample clustering is based on leading log2 fold changes. The primary axis explains 94% of the overall variance, cleanly separating the PBS replicates (blue) from the TGF-β1 replicates (red).

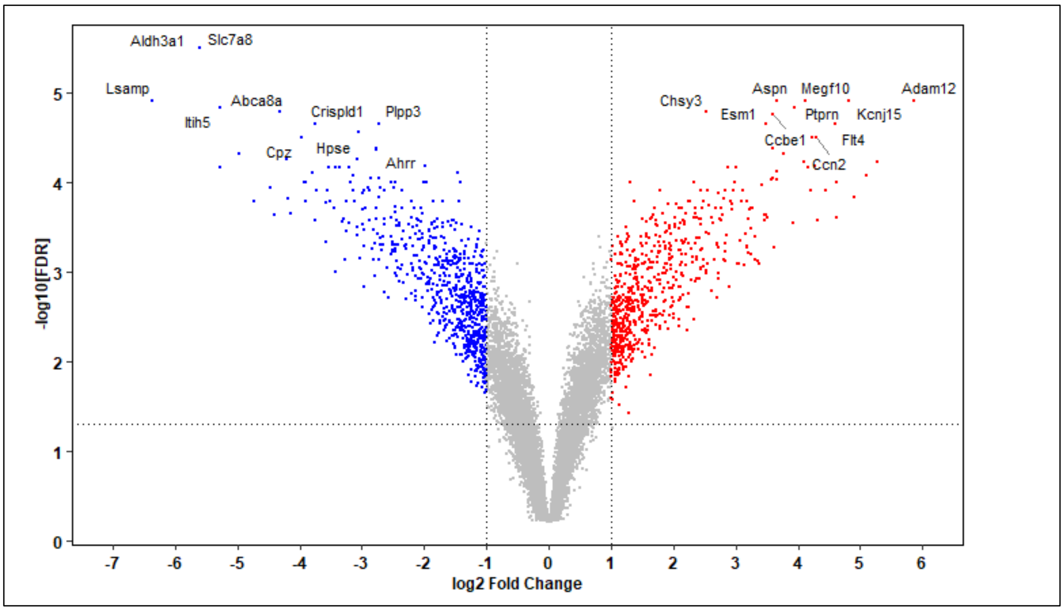

Supplementary Figure S3. Differentially Expressed Genes Volcano Plot. Statistical significance (-log10[FDR]) plotted against log2 fold change for all genes. Dotted lines define the screening thresholds (|log2FC| ≥ 1.0, FDR < 0.05). High-confidence upregulated DEGs are shown in red (678 genes) and downregulated DEGs in blue (712 genes), with key targets individually annotated.

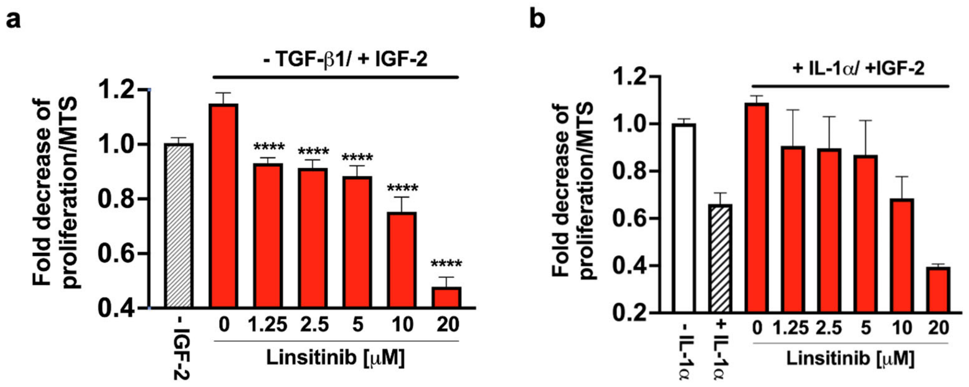

Supplementary Figure S4. Linsitinib suppresses IGF-2-mediated corneal myofibroblast survival under pro-apoptotic conditions.

(a) Dose-dependent inhibition of IGF-2-mediated corneal myofibroblast survival by linsitinib following TGF-β1 withdrawal. Corneal myofibroblasts were maintained in the presence of IGF-2 (100 ng/mL) and treated with the indicated concentrations of linsitinib. Cell survival was assessed by the MTS assay. **(b)** Dose-dependent inhibition of IGF-2-mediated corneal myofibroblast survival following IL-1α-induced apoptosis. Corneal myofibroblasts were treated with IL-1α in the presence of IGF-2 (100 ng/mL) and the indicated concentrations of linsitinib. Cell survival was assessed by the MTS assay. Data are presented as mean ± SEM (n = 6 independent experiments). Statistical significance was determined using one-way ANOVA followed by Tukey's multiple-comparison test. ****$P < 0.0001$ versus the IGF-2-treated control group.

Supplementary Table S1.

IGF-1	Forward	5′- GGAGGAGTTCGAGACAGAGTA-3′
	Reverse	5′-CGATGCGGTACAGAGTGAAA-3′
IGF-1R	Forward	5’- TGGTGGATGCTCTTCAGTTC-3’
	Reverse	5′- CTCATCCACAATGCCTGTCT-3’
Col1a1	Forward	5’- AGACCTGTGTGTTCCCTACT-3’
	Reverse	5’- GAATCCATCGGTCATGCTCTC-3’
Fbln2	Forward	5’- CGGGCAGAAGAAGACATAGAC-3’
	Reverse	5’- TGCACAACAGGTCTCGATTAG-3’
	Forward	5’- CCTTCCGCTGTGTAAATACCT-3’
	Reverse	5’- CTGCACACTCATCCTCATCTT-3’
GAPDH	Forward	5′-GGAGAAACCTGCCAAGTATGA-3′
	Reverse	5′-TCCTCAGTGTAGCCCAAGA-3′.
